# The Microbiome’s Influence on Head and Neck Cancers

**DOI:** 10.1007/s11912-022-01352-7

**Published:** 2023-01-25

**Authors:** Karolina Dorobisz, Tadeusz Dorobisz, Tomasz Zatoński

**Affiliations:** 1 Department of Otolaryngology, Head and Neck Surgery, Wrocław Medical University, Borowska 213, 50-556 Wrocław, Poland; 2 Department of Vascular and General Surgery, Wrocław Medical University, Borowska 213, 50-556 Wrocław, Poland

**Keywords:** Head and neck cancer, Microbiome, Microbiota, Oncogenesis, Radiotherapy, Chemotherapy, Immunotherapy, Toxicity

## Abstract

**Purpose of Review:**

Head and neck tumors (HNC) rank sixth among cancers worldwide. Due to their late diagnosis and poor prognosis, they are a clinical challenge. However, recent years have seen a dynamic development of science on the microbiome. The aim of the study is to discuss the role of the microbiome in HNC, the impact of the microbiome on oncogenesis, the course of the disease, as well as on treatment, and its toxicity.

**Recent Findings:**

The microbiome’s influence on oncogenesis, the course of the disease, and the effectiveness of oncological treatment have been confirmed in cancers of the colon, pancreas, lungs, and prostate. There is no solid literature on HNC. Many studies indicate disruption of the oral microbiome and periodontal disease as potential cancer risk factors. Disruption of the microbiome increases radiotherapy’s toxicity, intensifying radiation reactions.

**Summary:**

The microbiome plays an important role in cancer. It is a new target in research into new therapies. It may also be a prognostic marker of cancer development. Changes in the composition of the microbiome modulate the effectiveness of oncological treatment. More research is needed on the microbiome and its effects on HNC.

## Introduction

The incidence of head and neck cancer (HNC) globally ranks sixth, with over half a million new patients annually [1]. Men suffer from HNC more often than women: 5.8/100,000 to 2.3/100,000. HNC is a heterogeneous group of neoplastic diseases with significant differences between the European and US populations in terms of incidence and survival [2, 3]. The 5-year survival rate in HNC is below 50% [4, 5]. The most common cancer among HNC is laryngeal cancer; the prevalence in the EURoCare-5 population was 4.6/100,000 [3]. Research shows that infectious agents cause 15 to 20% of cancers, 20 to 30% are caused by smoking, and 30 to 35% are caused by lifestyle, improper diet, lack of physical activity, and obesity [6]. The main risk factors for HNC with a well-known mechanism of carcinogenesis are smoking, alcohol consumption, and the human papillomavirus (HPV), primarily type 16, and the Epstein-Barr virus (EBV). Oncogenic viruses have been well studied and described. HPV-based HNCs have different clinical characteristics and a better prognosis [7]. EBV detected in nasopharyngeal neoplasm significantly worsens the prognosis [8, 9]. Oncogenic viruses integrate viral DNA into the host genome [10] and inactivate tumor suppressor genes like p53 [11]. Despite combined treatment methods based on surgery, radiotherapy, chemotherapy, and immunotherapy, 5-year survival in HNC varies between 25 and 60% [3].

The microbiome is all the micro-organisms found in a tissue, organ, or the entire body. It was first described in 2001 [12]. The microbiome includes genes and genomes of the microbiota, as well as products of the microflora and the host, such as plasmid DNA, viruses, archaea, and fungi [13]. The human microbiome is currently undergoing numerous analyses; its impact on the incidence of many diseases, including cancer, is being assessed. The human microbiome consists of over 100 billion organisms, mainly found on mucous membranes, giving 2 kg of mass [14, 15]. Due to the very important role it plays in the pathophysiology of human diseases, it has been called “the last human organ under active research” [16] and “the second brain” [17]. The human microbiome is individually variable; the greatest influence on its composition is exerted by environmental factors and the host organism [18, 19]. Smoking tobacco alters the lung microbiome [20]. A high-fat diet causes dysbiosis with the dominant *Fusobacterium nucleatum*, considered to be an oncogenic bacterium [21]. Moreover, drugs such as metformin and proton pump inhibitors alter the gastrointestinal microbiome [22]. The composition of the microbiome has been defined by the 16S ribosomal RNA (rRNA) gene for bacteria [23].

The microbiome imbalance is dysbiosis, which is usually associated with diseases. Dysbiosis is primarily associated with intestinal inflammation, obesity, and allergies. It also occurs in multiple sclerosis, autism, depression, as well as in cancer [24–26]. It is believed that the pathogenic microbiome promotes oncogenesis through mucositis and general disorder of the body’s metabolism. The impact of the microbiome on the immune system function is particularly important [27, 28]. The analysis of over 1500 tests showed that tumor microbiome is mainly intracellular bacteria [29]. Extracellular microorganisms that are a microflora of intestines, mouth, vagina, or skin also have a very important function [30–32], and also affect oncological treatment [33].

## Study Design

The purpose of the work is to discuss the role of the microbiome in HNC, the influence of the microbiome on oncogenesis, the course of the disease, as well as on treatment and its toxicity. The literature search strategy was carried out using the Web of Science and PubMed base based on the keyword combination: Head and Neck Cancer, Microbiome, Microbiota, Oncogenesis, Radiotherapy, Chemotherapy, Immunotherapy, Toxicity. Studies were excluded if any of the concepts included in the search were not central to the article or if they used culture-based methods.

## Microbiome Analysis Technologies

Microbiome analysis technologies are based mainly on metagenomic bacterial sequencing of 16 s RRNA and shotgun sequencing. 16 s RRNA sequencing allows for the quantitative assessment of bacterial composition, assessing their type [34, 35]. Shotgun sequencing allows you to assess the composition and number of microorganisms [36]. The new technology is also IS-PRO, where the S-13 RRNA gene regions with lengths specific to each species of microorganism are amplified [37]. Analyzing MRNA, on the other hand, shows the activity of organisms, but only at the time of the study, it is not possible to determine the long-term relationships based on it, but only gene expression when the sample is taken [38]. Other techniques include the assessment of proteins or other metabolites, which include fatty acids, bile acids, and vitamins. Thanks to this technology, we are able to assess the effect of the microbiome on diseases. This opened the path to new diagnostic and treatment opportunities, giving hope for new anti-cancer therapies, especially in HNC, where treatment and survival of patients is unsatisfactory.

## Influence of the Microbiome on Neoplastic Diseases

The first reports of the microbiome and oncogenic or pathogenic microbiome date back to the beginning of the twenty-first century. The intensive development of science concerning the microbiome allowed for the evaluation of its influence on cancerous diseases. Neoplastic tissue is characterized by intense inflammation; the disorder mainly affects the immune system. Uncontrolled inflammation leads to hypoxia and necrosis, which facilitates the growth of anaerobic bacteria [39]. Inflammation in neoplastic disease has been known for many years, especially in HNC and neoplasms of the gastrointestinal tract, where the rich flora of the upper respiratory and gastrointestinal tract allows for uncontrolled bacterial growth in a favorable environment.

The metabolites produced by the host organism and its microbiome are modulators of many processes, maintain homeostasis, maintain proper metabolism, stabilize inflammation, and proliferation [40•]. They can change the microenvironment of tissues, especially cancerous tissues. Bacteria activate the immune system, can increase the number of neutrophils, and upregulate oncogenic pathways. Bacteria accelerate neoplastic transformations in patients with genetic susceptibility to cancer [41]. First of all, the reduction in the diversity of bacterial species is worrying; the predominance of one or more pathogenic species may be the beginning of cancer development.

The above information allowed us to conclude that the tumor microbiome and the surrounding tissues differ significantly from the microbiome of healthy tissue. The microbiome in neoplastic diseases not only changes the flora of the tumor and the surrounding area, it can move through the blood along with neoplastic cells to distant organs and systems, similarly to distant metastases [42]. The oncogenic microbiome may cause dysregulation of the entire body’s metabolism. The same types of bacteria were found in the upper respiratory tract and the upper gastrointestinal tract as in esophageal neoplasms or cervical cancer, these relationships have not yet been elucidated, but this confirms the influence of the microbiome on all human tissues [43, 44].

The microbiome has the greatest influence on the mucous membranes and richly colonized tissues. The digestive system is home to the most bacteria. *Helicobacter pylori*, a bacterium recognized as oncogenic, contributes to the development of gastric cancer by increasing the expression of cyclooxygenase 2 (COX-2), cytokines, and reactive oxygen species, thus the bacterium induces DNA oxidative damage, promoting oncogenesis [45]. As a result, *Helicobacter pylori* causes about 5% of cancers in the world [46].

The advantage of pathogens such as *Fusobacteria*, *Providencia*, and *Actinobacter* in neoplastic tissue has been confirmed in colorectal neoplasms [47, 48]. Many scientific reports confirm the oncogenic effect of these bacteria on the mucosa; moreover, they increase staging and grading of the tumor and the response to chemotherapy is much worse [49, 50]. In their study, Bullman et al. [51] presented the effect of metronidazole on the reduction of the microbiome and inhibition of colon cancer growth. Intestinal dysbiosis also reduces the amount of protective butyrate and contributes to an increase in cell proliferation. Changes in the gut microbiome also affect other cancers, and a decreased response to immunotherapy has been observed in kidney cancer, melanoma, and lung cancer in people with dysbiosis. This is confirmed by the fact that the gut microbiome influences the immune system.

The microbiome in patients with lung cancer differs from that in patients with chronic lung disease, with *Streptococcus* and *Prevotella* dominating in cancer [44]. According to Lee et al. [52], *Veillonella* and *Megasphaera* can be considered as biomarkers for lung cancer. In pancreatic cancer, the microbiome is the same as in the intestines—it is suspected that the microbiome may change the pancreatic microenvironment, leading to oncogenesis [53]. Changes in the microbiome have also been confirmed in breast cancer, where the bacteria present in the cancer are different from those found in normal tissue [54]. Proteobacteria, Actinobacteria, and Firmicutes are found in breast cancer and the surrounding tissue. Brewster et al. [55] found microbiome changes in female genitals that may be associated with ovarian cancer and endometriosis. In prostate cancer, the presence of pathogenic bacteria such as *Escherichia*, *Mycoplasma*, and *Cutibacterium* has been confirmed [56]. Studies on mice have shown that implantation of *Escherichia coli* and *Cutibacterium acnes* after prostatectomy induced inflammation and prostate adenocarcinoma [57.58]. In bladder cancer, the amount of *Fusobacterium* and *Campylobacter* is increased [59]. In the described examples, it was confirmed that the neoplastic tissue has a modified microbiome that can be considered pathological.

## Influence of the Microbiome on Head and Neck Cancers

Many cancers arise from inflammation, and it has been confirmed by HNC that smoking and alcohol consumption induce inflammation that leads to cancer formation. Research has confirmed that the microbiome can also promote inflammation and oncogenesis. The microbiome promotes inflammation in the airway epithelium in chronic obstructive pulmonary disease, cystic fibrosis, and asthma [60]. The interaction of the microbiome is synergistic with that of alcohol; bacteria metabolize ethanol, mediating the formation of acetaldehyde, which is a highly toxic compound. Acetaldehyde interferes with DNA synthesis and repair, increasing the risk of HNC [61]. Bacteria present in saliva—*Streptococcus salivarius*, *Corynebacterium*, and *Stomatococcus* have strong oxidizing properties, which allows for intensive metabolism of ethanol [62]. The tissue adjacent to the tumor may have the most significant impact on oncogenesis—its dysbiosis causes changes in the functioning of the immune system, which makes it possible to oncogenesis. Most of the literature is about the oral microbiome, which is the richest in the entire human body. There are 770 species of microorganisms in the oral cavity. Their composition is influenced by hygiene, smoking, diet, and alcohol consumption [63, 64]. Mukherjee et al. [65], Yost et al. [66], and Yang et al. [67] noted that in patients with oral cancer, dysbiosis enhances tumor growth. *Porphyromonas gingivalis* has the ability to stimulate oncogenesis in the oral cavity [68]; high levels of class G antibodies in the serum against this bacterium found in patients with gastrointestinal cancer and HNC [69]. Patients with diagnosed *Porphyromonas gingivalis* in the oral cavity have higher mortality [69]. This bacterium stimulates the production of myeloid-derived dendritic suppressor cells, which inhibit cytotoxic T lymphocytes, also induce overexpression of pro-matrix metalloproteinase-9 and reduce TP53 expression, thus inducing cell proliferation [70, 71].

The increase in mortality due to HNC is also significant with accompanying periodontitis [72]. *Streptococcus anginosus* in dental plaque increases the synthesis of nitric oxide and cyclooxygenase-2, increasing the risk of DNA damage [73]. However, no such correlation was observed in caries. Gong et al. [74] found the advantage of *Fusobacterium* in cancer of the larynx and surrounding tissue. The microbiome of the upper respiratory tract also affects the lower respiratory tract; dysbiosis in this area contributes to the development of lung cancer—the presence of the same pathogenic bacteria in the oral cavity and lung tumor tissues in patients with lung cancer has been confirmed [75]. On the surface of oral carcinomas, anaerobes such as *Actinomyces*, *Clostridium*, *Fusobacterium*, *Porphyromonas*, and *Bacteroides* are found. *Candida albicans* is the dominant among fungi, as well as aerobic bacteria such as *Klebsiella*, *Citrobacter*, *Streptococcus*, *Enterobacter*, and *Serratia* [76]. Frank et al. [77••] confirmed that *Lactobacillus* abundance is increased and *Neisseria* decreased in HNC. They also confirmed that reducing the pathogenic microbiome inhibits HNC oncogenesis, while transfer of microflora from HNC mice accelerates oncogenesis. It has been suggested that the administration of antibiotics prevents or delays the induction of the Ahr pathway [78]. AhR activation is a very important pathway in neoplastic diseases [78]. On the other hand, Dou et al. [79•] observed that increased numbers of *Schlegelella* and *Methyloversatilis* in HNC are associated with poor prognosis, while the dominant *Bacillus*, *Lactobacillus*, and *Sphingomonas* are found in patients with favorable prognosis. The oncogenic effect of *Fusobacterium nucleatum* in oral cancer has also been confirmed; it interacts directly with epithelial cells, causing faster growth and easier spread of the tumor [80]. This bacterium also inhibits apoptosis, thereby stopping the attempt to eliminate damaged cells. Little is found in the literature on the correlation of the microbiome with laryngeal cancer. Gong et al. [74] confirmed significant differences in the microbiome and recognized bacteria as a potential carcinogen. *Haemophilus influenzae* may also promote oncogenesis by IL-17 and neutrophil infiltration [81].

HPV-positive HNC have different clinical characteristics than other cancers; they occur in younger, not smoking, not drinking alcohol patients; their prognosis is better; and they are usually detected at an early stage [82]. Oncogenic bacteria can also change the course of cancer; however, to indicate these relationships, further scientific research is needed.

The microbiome can also support the fight against cancer or prevent them, *Corynebacterium* and *Kingella* change the risk of HNC by biodegradation and metabolism of poisonous substances such as toluene, styrene, and chlorobenzene. *Actinomyces* can reduce the risk of throat cancer, while *Neisseria sicca* can reduce the risk of oral cancer.

## The Role of the Microbiome in Cancer Therapy

The microbiome can influence the effectiveness of immunotherapy, chemotherapy, and radiation therapy. In patients with metastatic melanoma treated with anti-CTLA4 ipilimumab, microflora enriched with Firmicutes and *Faecalibacterium* gives longer progression-free and overall survival than in microbiota dominated by *Bacteroides* [83]. *Bifidobacterium* also supports melanoma therapy [84]. According to Routy et al. [85], patients resistant to immunotherapy are mainly people with dysbiosis and those who have undergone antibiotic therapy. Patients immediately after antibiotic therapy have a greater risk of rapid disease progression in lung cancer [86].

The microbiome also influences the effectiveness of cisplatin and cyclophosphamide chemotherapy [87, 88]. Modifying the microbiome, reducing pathogenic bacteria supports the effectiveness of immunotherapy and chemotherapy, and reduces the side effects of this treatment.

Radiotherapy is a very important method in the treatment of HNC. Radiotherapy can be used as a radical treatment or in combination with chemotherapy as adjuvant or palliative treatment [89•]. The effect of radiation therapy is to damage cancer cells, but also to damage the cells surrounding the cancer. Radiotherapy causes side effects such as stomatitis, xerostomia, dysphagia, odynophagia, and chronic sinusitis [89•, 90, 91]. These symptoms have a significant impact on the quality of life of patients, and sometimes even lead to the discontinuation of oncological treatment. Intensive research is aimed at reducing the toxicity of radiotherapy treatment. One potential approach is to modify the microbiome to protect the mucous membranes. The radiation induces inflammatory processes, leading to apoptosis or cell death. Necrotic tissue is formed at the site of intense radiation, which promotes dysbiosis and uncontrolled multiplication of bacteria. Microbiome homeostasis during radiotherapy depends on the number of bacteriophages, the number of which also declines due to radiation. Commensal bacteria can become pathogenic, and the growth of pathogenic bacteria can lead to serious inflammatory complications. Stokman et al. [92] assessed the effect of topical broad-spectrum antibiotics on the microbiome and the severity of stomatitis after radiotherapy and found that it had no effect and did not alleviate the symptoms of the disease. Antibiotic therapy may have a negative effect on the dysbiosis during radiotherapy. Studies have confirmed that the diversity of the microbiome decreases with the radiation dose rate [93, 94]. The lower diversity of bacteria causes dysbiosis and the uncontrolled multiplication of pathogenic bacteria.

Over 90% of patients with HNC develop stomatitis after radiotherapy, and 60% of them have inflammation classified as severe accompanied by complete dysphagia [90]. Dysbiosis promotes the persistence of ulcers and delays healing [95•]. *Actinobacillus*, *Mannheimia*, and *Streptobacillus* are associated with increased severity of oral mucositis [96]. *Fusobacterium* and *Haemophilus* dominant in the oral microbiome before radiotherapy are associated with susceptibility to inflammatory complications. *Prevotella*, *Fusobacterium*, and *Streptococcus* are considered as prognostic biomarkers of the onset of oral mucositis, while *Megasphaera* and *Cardiobacterium* are biomarkers of severe inflammation [95•]. In a study by Jiang et al. [97], it was shown that patients who received probiotics during chemoradiotherapy developed less oral mucositis compared to the group without probiotics in therapy (15.5% vs. 45.7%). A study by Ma et al. [98] showed that patients with probiotic therapy more often undergo radiotherapy without complications, compared to the group without probiotics in therapy, where patients discontinued treatment due to complication. Late complications of radiotherapy include tumors induced by oncological treatment, especially after HNC treatment, one of the causes may be persistent dysbiosis of the upper respiratory tract.

The microbiome can be modified by symbiotic treatment, using prebiotics and probiotics. *Bifidobacterium* improves the course of diabetes and allergies. According to Rong et al. [99], the intake of *Lactobacillus helveticus* may suppress hyperplasia and oncogenesis by reducing the number of T lymphocytes. In patients with dysbiosis and lack of improvement of flora with symbiotic treatment, the use of antibiotics may also be effective. Maintaining a normal microbiome in the body is a way to avoid cancer. In a study conducted on mice, probiotics used in inhalation strengthened lung resistance to metastases [100].

## Limitations of the Study

Many publications are based on small study groups. The microbiome is influenced by many environmental factors that should be thoroughly assessed. Due to the ongoing research on the microbiome, we should be very critical of knowledge on this subject and select only publications with a large study group and an appropriate control group.

## Conclusions

HNC is a multifactorial disease; environmental factors influence its development, course, and treatment.

The differences between the microbiome of healthy people and HNC patients have been confirmed.

The microbiome can be oncogenic by intensifying the inflammation of the mucous membranes, systemic effects, as well as changes in anti-cancer resistance or by inhibiting the effectiveness of cancer therapy.

The presence of HNC drives changes in the microbiome or dysbiosis induces oncogenesis.

The upper respiratory tract dysbiosis with smoking and alcohol consumption is an important HNC risk factor.

Bacteria can be a prognostic biomarker of HNC development.

The interaction of the dysbiosis with immunotherapy, chemotherapy, and radiotherapy affects the effectiveness of treatment and the intensification of the side effects of these therapies.

Many clinical trials about the microbiome and dysbiosis are still at an early stage, but there is enormous potential to use it in the future.

A targeted therapy against the microbiome of cancer can contribute to HNC prevention, inhibiting cancer progression or increasing the effectiveness of treatment.
